# Defining futile life-prolonging treatments through Neo-Socratic Dialogue

**DOI:** 10.1186/1472-6939-14-51

**Published:** 2013-12-09

**Authors:** Kuniko Aizawa, Atsushi Asai, Seiji Bito

**Affiliations:** 1 Office for Research Ethics and Bioethics, Research and Development Initiative Center, National Cerebral and Cardiovascular Center, 5-7-1 Fujishiro-dai, Suita-shi, Osaka 565-8565, Japan; 2 Department of Bioethics, Faculty of Life Sciences, Kumamoto University, 1-1-1 Honjo, Chuo-Ku, Kumamoto-shi, Kumamoto, 860–8556, Japan; 3 Division of Clinical Education and Division of Clinical Epidemiology, National Hospital Organization Tokyo Medical Center, 2-5-1 Higashigaoka, Meguro-ku, Tokyo 152-8902, Japan

**Keywords:** Life-prolonging treatment, Futility, Socratic Dialogue, Philosophical group discussion

## Abstract

**Background:**

In Japan, people are negative towards life-prolonging treatments. Laws that regulate withholding or discontinuing life-prolonging treatments and advance directives do not exist. Physicians, however, view discontinuing life-prolonging treatments negatively due to fears of police investigations. Although ministerial guidelines were announced regarding the decision process for end-of-life care in 2007, a consensus could not be reached on the definition of end-of-life and conditions for withholding treatment. We established a forum for extended discussions and consensus building on this topic.

**Methods:**

We used the Neo-Socratic Dialogue (NSD) method which promotes philosophical discussion based on a case-study to address a question and formulate a consensus and answer in a group. The question chosen for the dialogue was: “What is a life-prolonging treatment?” A series of dialogues took place over a period of one and a half days. It was carried out by three groups in 2010 and 2011. Seven participants with diverse backgrounds were recruited per group. We analyzed the content of the discussion.

**Results:**

Based on three case studies concerning different opinions about treatment options for an older dementia patient, a patient demanding chemotherapy, and a severely ill neonate, conditions for futile life-prolonging treatment were elucidated through NSD. Such treatments are those carried out for the sole purpose of prolonging life and are detrimental to the patient, and should be decided based foremost on the patient’s lack of desire for treatment, the consensus of those involved, and through social acceptance. These arguments are essentially consistent with ones on medical futility in the United States. By expressing the objective of healthcare and the requirement of social acceptance, participants were also able to elucidate issues related to the awareness of those involved and the medical environment. Compared to the end-of-life guidelines in Japan, the objective of treatment, its effects, and benefits were more specifically discussed with the patient’s intentions as the foremost consideration, rather than being limited to the terminal stage.

**Conclusions:**

This small study contributed to elucidating the conditions and current problems of futile life-prolonging treatment through NSD. They would suggest more substantial guidelines and improvements on the administration of the treatment.

## Background

Since the latter half of the 1970s, movements related to euthanasia, death with dignity, and hospice care have largely criticized life-prolonging treatments [1,2]. Indeed, a survey conducted in 2008 by the Ministry of Health, Labour, and Welfare (MHLW) regarding interest in end-of-life care revealed that more than 70% of responses were negative toward life-prolonging treatments when “death is imminent within a few days,” “death is imminent in approximately 6 months,” “there is persistent disturbance of consciousness,” or “the patient suffers from cerebrovascular disease or dementia.” [3] Against this backdrop, the number of members of the Japan Society for Dying with Dignity recently surpassed 120,000, or roughly 0.1% of the Japanese population. The Society’s stance is to refuse life-prolonging treatments that solely aim to delay death in cases of incurable or terminal disease. In the event of persistent disturbance of consciousness (i.e., persistent vegetative state), they advocate the discontinuation of life-support measures [4].

In 2005–2007, a number of cases involving the removal of respiratory apparatuses were investigated by police and widely reported by the media, although the charges were later dropped [5]. These incidences prompted the MHLW Investigation Committee to publicly announce guidelines regarding the decision process for end-of-life care in 2007. Despite these efforts, a consensus could not be reached on the definition of “end-of-life” and criteria for discontinuing treatment. Thus, final judgments and decisions are left to the discretion of the healthcare team [6-8]. Although laws that regulate withholding or discontinuing life-prolonging treatments and advance directives do not exist in Japan, physicians view discontinuing life-prolonging treatments negatively due to fears of police investigations. This situation underscores the need for extended discussions on this topic. In other countries, particularly the United States, medical futility and the question of whether physicians should unilaterally make decisions about withholding or discontinuing life-prolonging treatments were being debated from about 1990 [9]. Debates to this degree have yet to be carried out in Japan [10].

Changes and diversification of public values lead to the development of a wide range of views focused on the basic objectives of healthcare. Few opportunities exist for professionals and patients to discuss healthcare issues and concerns. Nevertheless, these issues need to be addressed with a particular focus on the future of Japan’s healthcare system. Exploring these concerns is vital, and efforts are needed to create and test a communication model that aids in mutual understanding and consensus building. The Neo-Socratic Dialogue (NSD) method can be used in forums focused on issues, principles, and values related to healthcare among participants of diverse backgrounds. In the present study, NSD was used to address the question “what is a life-prolonging treatment?” This small study contributed to elucidating the conditions and current problems of futile life-prolonging treatment through NSD.

## Methods

NSD promotes philosophical dialogue among small groups of approximately seven people. This method was formulated by Leonard Nelson (1882–1927), and is presently used in Germany, England, and Holland for philosophical training, dialogue-based education, problem discovery, and for establishing consensus [11,12]. In 1999, the method was introduced in Japan and has been in use ever since. Recent attempts have been made to apply NSD to ethical and social discussions spanning the medical and healthcare fields [13-17].

NSD typically starts with a general question. Each participant then provides relevant case studies, and one is selected for further investigation and discussion. Participants corroborate judgments and actions taken by those involved in the selected case study, and abstract the underlying principles and values. Finally, a consensus is formulated around the initial question. To ensure the success of any given dialogue, participants are asked to conform to specific rules, which include speaking clearly and listening carefully to other participants. Participants are also provided with appropriate standards to help select a case study suitable for dialogue.

In the present study, a series of dialogues were divided into six 90-minute sessions that took place over a period of one and a half days. These sessions were carried out by three groups in 2010 and 2011 in Tokyo, Osaka, and Kumamoto, which are major cities in eastern, western, and southern Japan. The general question chosen for the dialogues was: “What is a life-prolonging treatment?” While the term “life-prolonging treatment” is used in herein, “life-prolonging therapy” and “life-prolongation” were also used in the forums. Seven participants were selected per group and recruited among acquaintances of researchers that contributed to the study. The following participants were enlisted in each group to illustrate different standpoints on the question: two participants representing the general public; an ethicist or bioethicist; a participant with a legal background, a participant representing the mass media, or a sociologist; a physician; a nurse; and an additional healthcare professional. When a participant could not found, however, willing volunteers from any background were invited. We also attempted to recruit so that each group had at least three men and three women. In addition, we anticipated that various types of case studies would be selected and examined in the three forums. During the discussions, a facilitator wrote the main points on a flip chart, while a transcriber recorded participant statements using a computer terminal. The discussions were also recorded on an IC recorder with participants' agreement to supplement these documents.

The content of the discussions were reviewed and analyzed from the following standpoints that had normally been followed in NSD: what kinds of views were presented?; what were the reasons of the different views?; what kinds of principles and values were revealed?; were there any conflicts among them?; and what consensus and answers were elucidated ?

This study was approved by a General Research Ethics Review under the auspices of the Faculty of Medical and Pharmaceutical Sciences at Kumamoto University (Ethics No. 282 < modified>, issued March 31, 2010). Participants received written information about the study and provided written informed consent to participate.

## Results

The total number of participants for the three forums was 19, with a total of 40 case studies provided (see the ‘List of participants’ and ‘List of case studies’ sections). The origin of the case studies included hospitals (24 cases), home (6), others (4), and unspecified (6).

### List of participants

Total participants for all three forums: 19 (5 men, 14 women)

The 1^st^ forum (July 2010, Tokyo): 6 (1 man, 5 women)

Participants: 2 editors, lawyer, physician, 2 nurses

The 2^nd^ forum (January 2011, Kumamoto): 6 (3 men, 3 women)

Participants: citizen (only on second day), bioethicist, media representative, psychiatrist, nurse, pastor

The 3^rd^ forum (February 2011, Osaka): 7 (1 man, 6 women)

Participants: citizen, ethicist, scientific writer, physician, 2 nurses, care manager

### List of case studies

40 case studies: palliative care (7 cases), treatment of elderly (4), long-term artificial respiration (3), family requests for treatment (3), family decisions (3), discussions among family members (2), family care (2), cancer chemotherapy (2), refusal of treatment (2), informed consent (1), advance directive (1), DNAR order (1), treatment of severely ill neonate (1), physicians’ flaunting of authority (1), family burden (1), psychiatric disorder (1), medical journalism (1), and pet euthanasia (1).

### The 1^st^ NSD forum

Thirteen case studies were provided regarding the question “What is a life-prolonging treatment?” The following case was chosen to explore the meaning that various medical treatments had for those involved in the case.

#### Case 1: Differing opinions regarding the treatment of elderly with dementia

##### Case source

Resident physician

##### Summary

One of my patients was a woman in her 90s who suffered from dementia and valvular disease. She lived with her eldest daughter, and her second daughter lived away from home. The eldest daughter called the hospital several times regarding the patient’s chest pain, and after visiting the cardiovascular internal medicine department, she was admitted to the hospital. At admission, she presented with a fever and poor respiratory status. Complication by pneumonia was suspected. The patient complained of pain and was in agony, but was not competent to consent to treatment. The attending physician at the cardiovascular department initiated intravenous drip and diuretics to treat heart and respiratory failure, but did not use anti-microbials, which was incomprehensible to me. On day 2 after admission, she presented with arrhythmia (atrial fibrillation) and her blood pressure decreased. Although an anti-arrhythmic agent was administered, it was ineffective. Given the state of emergency, defibrillation (DC) was attempted once without obtaining consent from the family. Immediately afterwards, the attending physician proposed Do Not Attempt Resuscitation (DNAR) to the family because a full recovery was unlikely given the patient’s age, as well as to prevent further pain from DC or artificial respiration. The attending physician seemed to negatively view life-prolonging treatments. Although the eldest daughter desired to prolong her mother’s life to the greatest extent possible, the second daughter differed in opinion, feeling sorry for her mother and not wanting her to suffer any longer. Because the patient presented with refractory arrhythmia, she was anesthetized to relieve pain, and DC was continued while rapidly increasing the voltage. At times, DC was carried out multiple times a day, totaling about 20 times over the span of a few days. Subsequently, intratracheal intubation was decided against, but DC was continued and administration of a strong anti-arrhythmic was initiated. One week after hospitalization, arrhythmia resolved somewhat and DC became unnecessary. This is when the decision for DNAR, including that related to DC, was decided on. Despite these improvements, pneumonia and respiratory discomfort worsened, and the patient had difficulty speaking. Although nutritional intake was achieved with intravenous drip, transfusion was difficult due to heart failure, leading to malnutrition. Despite the prohibition of meals, the eldest daughter fed her mother on several occasions. Alleviation of respiratory discomfort was considered but not carried out due to the risk of death from reduced blood pressure. The eldest daughter, who observed her mother gasping and suffering, strongly requested artificial respiration. After this, the patient responded to anti-microbials, and was freed from the artificial respirator 10 days later. The treatment strategy was discussed with the eldest daughter again, and she agreed to forgo the use of an artificial respirator. The patient recovered thereafter, and in accordance with the eldest daughter’s wishes, a gastrostoma was inserted. The patient suddenly died three days later due to suffocation from aspiration. This entire process spanned 9 weeks. If treatment subsequent to the second DC was not carried out, the patient was thought to have lived for only one week.

#### Actions and judgments, and their reasons

In this case, there were many opinions regarding life-prolonging treatments, and the case provider questioned what the attending physician and patient’s family considered to be life-prolonging treatments. The actions and judgments of those involved were organized below.

Attending physician: *Although I have somewhat negative views on life-prolonging treatments such as anti-microbials, artificial respirators, and DC, I do carry out treatments for heart failure to some extent, as it is my specialty.*

Patient: No capacity to make decisions.

Eldest daughter: *I know what my mother’s wishes are. I want her to live as long as possible, for my sake as well as her own.*

Second daughter: *I feel that DC and artificial respiration are painful for my mother, and I feel sorry for her.*

Resident physician: *I question why the attending physician didn’t use anti-microbials, and whether that represents a life-prolonging treatment. How about other treatments? What makes a treatment a life-prolonging treatment?*

Next, we organized what those involved considered to be life-prolonging treatments, as well as the underlying reasons.

•Anti-microbials were considered a life-prolonging treatment to the attending physician because, although he likely intended to begin treatment for heart failure before pneumonia, anti-microbials were non-essential and non-urgent. Other treatments carried out at hospitalization were emergency treatments for rescue and pain relief, so were not considered life-prolonging treatments.

•To the attending physician, the first DC was an emergency measure before the family’s consent for DNAR was obtained, so it was not a life-prolonging treatment. However, DCs subsequent to the second one only increased suffering, and since heart failure could not be completely cured or controlled and death was imminent, they were life-prolonging treatments. The use of a strong anti-arrhythmic agent when continuing DC after the twentieth time was not considered a life-prolonging treatment because it was a measure associated with less suffering than DC, and was used for pain relief.

•According to the second daughter, DCs subsequent to the second DC only prolonged suffering until death, so were life-prolonging treatments.

•However, to the eldest daughter, the treatments held promise up to the time that DC became unnecessary one week post-admission, and since the other treatments were for alleviating pain and curing the patient, they were not life-prolonging treatments.

•To the attending physician, regardless of the difficulty of curing the condition with an artificial respirator, once the patient is on the respirator, it cannot be easily discontinued due to legal concerns, so it is a life-prolonging treatment.

•To the second daughter as well, since the artificial respirator cannot cure her mother and prolongs her suffering until death, it is a life-prolonging treatment.

•Although the eldest daughter at first agreed not to intubate the patient one week post-admission, she strongly desired artificial respiration when the patient began to exhibit signs of respiratory discomfort. Since this measure relieves the patient from pain, she did not consider it a life-prolonging treatment.

•The anti-microbial agent used concurrently with the initiation of artificial respiration may not necessarily be a life-prolonging treatment in the sense that the eldest daughter strongly desired it. Yet, the attending physician himself likely considered it a life-prolonging treatment.

•The decision not to re-intubate once taken off the artificial respirator was likely because, to the eldest daughter as well, it would prolong the patient’s suffering. Thus, it is a life-prolonging treatment.

•To the eldest daughter, the gastrostoma was inserted to prevent pneumonia, allow for nutritional intake, and for discharge from the hospital, and was thus considered a treatment to cure her mother’s condition. However, to the second daughter, since it was an unnecessary invasive treatment at the terminal stage, it was a life-prolonging treatment. The attending physician did not necessarily see it as a life-prolonging treatment, given the possibility of home care in a state of reduced activity.

Since the effects of treatment and chances of death were uncertain, participants indicated that whether a treatment is life-prolonging should not be judged based on the end result, but rather according to the intentions behind carrying out the treatments. They also noted the troubles arising from the different meanings the treatments had to those involved, such as rescue, life-prolonging, pain relief, curing, and prevention.

#### Life-prolonging treatment

Based on the discussion above, participants summarized the conditions for life-prolonging treatments as follows:

•A treatment that is carried out regardless of the possibility of healing and death is imminent (although there is some uncertainty).

•Suffering is severe.

•When patients consider the treatment to be futile themselves, and the family and healthcare team agree.

•When its futility is socially accepted.

Participants observed that decisions were influenced by the family’s opinions and opinions of others that comport with the social status of patients. With advances in life-prolonging technology, it is also becoming clear that there are situations when such treatments are futile at the terminal stage of an elderly person’s life. Yet, in the past, both society and physicians were of the mindset that life-sustaining treatments should be performed until the very last moment. In this case, given that the opinions of the patient, the family, and healthcare professionals differed, and that a stable social awareness was not achieved, participants indicated the need to increase social recognition of what futile life-prolonging treatments are. Although therapy generally aims to stop the worsening of a condition and improve it, this is not the full picture. Instead, therapy also encompasses emergency aid, pain relief, prevention, and life-prolonging for the sole purpose of preventing death. Thus, at some point, emergency aid procedures may be considered life-prolonging procedures. Although not recognized by patients, families, and the general public, there are situations where life-prolonging treatments and palliative treatments are incompatible. Participants agreed that for each of these situations, the objective of a particular treatment must be clearly explained to those involved, and the therapeutic effects and potential side effects sufficiently discussed, with treatments carried out by maintaining an appropriate balance. It was also proposed that the phrase “life-prolonging treatment” should not be used lightly, and when carrying out such treatments, healthcare professionals should aim for social consensus building when it comes to withholding or discontinuing these treatments.

### The 2^nd^ NSD forum

In this group, 17 case studies were provided regarding the question “What is a life-prolonging treatment?” The following case was chosen to address the question of how healthcare professionals should proceed when a patient strongly desires treatment until the very end.

#### Case 2: A patient who strongly desires chemotherapy to the greatest extent

##### Case source

Nurse

##### Summary

The patient was a 50-year-old male with chronic leukemia. Onset was about six years ago, and he had been visiting the hospital ever since. He was eventually hospitalized for chemotherapy and thereafter continued the routine of repeated hospitalization and home care. While chemotherapy dramatically reduces the size of enlarged lymph nodes and the spleen, bodily damage also increases with dosage escalation. For the first few months, the patient recuperated at home, but this gradually decreased to one month, then to one week, and after about one year, he could not be discharged from the hospital. Although the patient knew that a complete recovery was unlikely, chemotherapy was having an effect, thus both he and his wife desired continued treatment. When the effects finally began to wane, and it became difficult to predict whether bodily damage or the curative effects would be greater, he was told that he had about one month to live if therapy was discontinued. To the patient, choosing to discontinue chemotherapy was tantamount to giving into death, and thus he desired, as a last resort, to undergo aggressive treatment in a sterile room. Although the attending physician and I doubted the wisdom in carrying out chemotherapy, the disease specialist suggested that the choice be left to the patient. Since the specialist’s opinion was prioritized over the attending physician’s opinion within the medical team, chemotherapy was initiated. Even among the nurses, there were differing views on whether the therapy should be carried out. The patient experienced delirium and panic due to limited freedom in the sterile room. Yet, the healthcare team, in collaboration with the psychiatric department and palliative care team, provided care to maintain the patient’s life, as well as care for the wife. The treatment was covered by insurance. We were also able to secure a bed in the sterile room without issues. One month after initiating treatment, the patient desired further treatment, although he could not leave the sterile room. However, this was no longer possible. Soon thereafter, he was transferred from the sterile room to a semi-sterile room and his symptoms were controlled. At one time, the patient asked “how much longer do I have?” Although the physician estimated about one month, he responded that it depends on the person. At first, the patient asked “is it 3 years?”, to which the physician replied “that’s difficult.” To this, the patient continued “1 year?” “half a year?” “3 months?” For each of these, the physician replied “that’s difficult.” After that, the patient stopped asking, and seemed to have accepted the fact that death was near. Although he no longer desired chemotherapy, he proclaimed “now that I’ve heard that, I feel relieved,” and that “I will try to bring about a miracle.” Regarding intratracheal intubation, a resuscitation technique that was risky given his enlarged cervical lymph nodes, the patient mentioned that since he could not decide himself, the family should be consulted to make the decision. Although his wife initially desired intubation, the patient’s sister supported his shaken wife, and they eventually decided on DNAR. The patient’s eldest son is independent and lives far away from home. The patient’s sister explained the father’s condition to his teenage child. Although the patient died a few weeks later, his wife continued to slap his face throughout the night telling him “don’t die yet.” In the end, the eldest son restrained his mother. I believe that such cases will increase with advances in chemotherapy.

#### Actions and judgments, and their reasons

The participants summarized the case provider’s judgment as follows: Although providing treatment as requested by the patient is not necessarily a bad thing, there remains the question of whether the treatment should actually be carried out.

Participants considered the reasons for this judgment as follows:

•The positive aspect is that treatment was carried out to the greatest extent possible to fulfill the patient’s wishes in accordance with his values.

•However, regarding the question of whether the treatment was appropriate, the patient may have been able to maintain a higher quality of life (QOL) if the treatment was not carried out, e.g., he may have been able to walk, go home, eat, and lessen the burden of going to and from the hospital.

•Carrying out an aggressive treatment confers the risk of “therapeutic death.”

•In other words, although the patient’s judgment comported with his values, it was not a rational judgment based on careful consideration.

•Regarding the option of forgoing chemotherapy, this option may not have been sufficiently explained to the patient, and there may have been the lingering thought that this option would be impossible to communicate to the patient.

#### Life-prolonging treatment

Participants discussed what they consider to be life-prolonging treatments, and their thoughts are summarized below:

•Although not curative, it delays death, in the short- or long-term.

•When the patient desires it, it cannot be considered “futile.”

•It is a phrase commonly used when the patient does not desire it, and when it seems futile or detrimental to the patient from a third person’s perspective.

Given that one of the participants asserted that cancer chemotherapy carried out as a non-curative treatment is considered a life-prolonging treatment, the participants discussed this issue and concluded that life-prolonging by chemotherapy can either be long-term or short-term (i.e., due to a sharp deterioration in the patient’s condition). Moreover, one might say that since the patient asked for a life-prolonging treatment because he desired non-curative chemotherapy until the very end, it was by no means “futile” because the patient was willing to fight the disease. On the other hand, the patient left the decision of resuscitation to his family, who decided against it. In this respect, it might be considered a “futile” life-prolonging treatment. In any event, it was not possible to examine whether therapy would be considered beneficial if the patient’s family desires it in this particular case.

Participants also argued that there were issues regarding discussions among healthcare professionals and the patient/family about treatment strategy. While the specialist presented a choice between treatment and no treatment, he did not recommend one over the other and left the choice to the patient. Given the limited time for discussions between the patient/family and healthcare team, the patient kept to his decision for treatment. In order for the patient and family to carefully consider options, in addition to a need to be receptive to their anxiety, participants discussed the paternalism exhibited by the physicians. That is, the physicians took on an excessive degree of responsibility by keeping the patient from thinking about the decision because they could not deal with the patient’s anxiety, or by holding back recommendations in order to avoid leading them to a particular decision. Nurses also had no leeway and team medicine was not being adequately performed. There was also a shortage of interview rooms, and consultation services within and outside the hospital were not being fully introduced to the patient and his family. Participants thus agreed that there was the inadequate psychological support and lack of coordination for obtaining appropriate informed consent and carrying out the best medical treatment.

### The 3^rd^ NSD forum

In this group, 10 case studies were provided regarding the question “What is a life-prolonging treatment?” We chose the following case to illustrate judgments made by a medical professional.

#### Case 3: Treatment of a severely ill neonate

##### Case source

Physician

##### Summary

Several years ago, a pregnant woman in her mid-20s visited the hospital due to threatened premature labor. Therapy was unsuccessful and cesarean section was performed. The infant was born at 23 weeks at less than 600 g, received resuscitation therapy, and was admitted to the NICU. The infant was kept in an incubator with artificial respiration and received hydration and nutrition drip infusion. While the survival rate of neonates in this condition is usually about 30%, it exceeds 50% at this hospital, so consent was obtained from the parents after assuring them that the fullest effort will be made with the aim of discharging the infant alive. Three days later, an ultrasound exam revealed minor intracranial bleeding, which worsened by day 4, at which hydrocephaly, disseminated intravascular coagulation (DIC), anemia, and reduced blood pressure became apparent. Although therapy was carried out with extensive transfusion and blood products, multiple organ failure and anasarca progressed, and by about day 10, these conditions were considered irreversible. This decision was based on test data, as well as the physician’s experience based on the infant’s body color and activity. While I wanted to shift from aggressive to palliative treatment, I could not get majority approval at a conference regarding treatment strategy from other physicians who had worked at the facility for many years, although the supervising physician approved. Thus, I could not discuss switching the treatment strategy with the parents. In my view, treatment that exceeds the healing capacity of the infant merely delays death, although there were also opinions that a clear line could not be drawn regarding what was considered excessive treatment. Physicians also differed in opinion with respect to how much blood to transfuse, and some thought that administering anti-microbial agents without transfusion was contradictory. In my view, it was not in the best interest of the patient and family to continue aggressive treatment until the infant worsened to a severely debilitated state. The parents were always able to visit the infant, and either the father or mother came once every day. I explained the infant’s condition to them every time, at times together with the supervising physician, but the parents did not give their opinions much or ask many questions. Explaining the specialized therapy to the parents was difficult, particularly because such explanations might convince the parents to discontinue treatment. Thus, based on the unilateral judgment of the physicians, treatment was continued, and the family did not have the opportunity to voice their opinions. This, to me, is what led to the life-prolonging treatment. On day 18, the infant presented with pneumonia and anti-microbial treatment was initiated, although it was already too late. It was explained to the parents that sudden changes were possible, but that treatment would be continued. On day 19, the infant’s condition suddenly deteriorated, and after limited chest compression, I explained to the parents the intent to discontinue treatment, and the infant died. The infant was discharged from the hospital to spend the final moments with the family. The infant did not have any interactions with grandparents.

Although other physicians thought that curing the infant’s condition was difficult, they said nothing, and when the infant died, they had the air of “so it didn’t work after all…” They considered aggressive therapy their duty, and given the difficulty of accepting death, they did not mention the option of discontinuing treatment. However, physicians should not only set their minds to the continuation of treatment, they should also think about the treatment’s objective.

#### Actions and judgments, and their reasons

The participants summarized the case provider’s judgment as follows: Not switching treatment strategy the moment the infant’s condition became irreversible is considered excessive treatment.

Participants considered the reasons for this judgment as follows:

•During the course of treatment, there was no medically-justifiable prospects for the infant‘s recovery.

•Continuing aggressive treatment is cruel to the patient because detrimental aspects far outweigh the benefits. In that sense, discontinuing aggressive treatment is not “giving up,” but rather is in the best interest of the patient.

•Physicians only discussed strategy among themselves and considered continuation of treatment to be the best course of action, but did not explain this to the parents or listen to their wishes.

The participants indicated that, although decision making regarding treatment would be very difficult for the family, the physicians only had aggressive treatment to the last moment in mind, and believed that this was in the family’s best interest. Yet, since different families have different values, the decision made by the physicians cannot be considered appropriate.

The participants also discussed what was in the best interest of the infant. At this stage, the infant exhibited biological responses to pain and respiratory discomfort. However, given that the nerves in the brain were still underdeveloped, it was unclear whether the infant could sense happiness and joy. It was also possible that being embraced by the parents may have had a positive influence. In sum, the participants concluded that although the patient may not have had intentions, the best interests of the patient should have been considered, keeping in mind the fact that the patient is a human being.

#### Life-prolonging treatment

Based on the discussion above, participants discussed what they considered to be life-prolonging treatments, and their thoughts are summarized below.

While life-prolonging treatment refers to a treatment that sustains life, a life-prolonging treatment is considered futile when:

•The body is incurable and edging towards death.

•Detrimental effects (pain and suffering) outweigh the benefits (healing and QOL) , and the patient does not desire the treatment.

•However, despite the points above, losing sight of the patient’s best interest, which is the objective of healthcare, and aiming solely to delay death.

•Moreover, a treatment that would be detrimental to the patient, even if it is pursued in consideration of the family’s intentions.

The case provider indicated that when aggressive therapy is the only objective, discontinuing it would be considered giving in. In this case, physicians will lose sight of the objective when the condition becomes incurable and the objective can no longer be accomplished. Participants also discussed the parent’s intentions and agreed that while the parent’s intentions should be considered, the patient’s best interests should be the main consideration.

## Discussion

The first forum discussed an elderly patient who lost the ability to make decisions, the second forum discussed a middle-aged patient who strongly desired treatment, and the third forum discussed a case involving a neonate. These case studies from hospitals illustrated various situations. We have summarized answers derived in the forums with respect to “what is a futile life-prolonging treatment” as discussed below.

### What is futile life-prolonging treatment?

•A treatment carried out, without a reason other than to prolong life, which is detrimental to the patient.

This judgment is made via the following process:

•Confirm that the patient does not desire it (refuses, has no intention to undergo treatment, or prior intention was unclear or does not exist),

•consider the family’s opinions,

•reach a consensus among the healthcare team, and

•obtain social acceptance.

This answer suggests that the decision of whether a life-prolonging treatment is futile is made based on the purpose of treatment, whether it is effective, and the extent of its benefits. This judgment also incorporates the patient’s intent, family’s intent, consensus among healthcare professionals, and social acceptance. This answer, discussions leading to the answer, and the significance of NSD, are discussed further below.

### Futile life-prolonging treatments

#### The purpose of treatment and its effects and benefits

First, we discuss the purpose of treatment, as well as its effects and benefits to the patient. The various objectives of treatment should be specified, and the final objective should be set as “the best interest of the patient.” NSD revealed that recognition among those involved was lacking. For instance, withholding anti-microbial agents in the first case and continuing aggressive therapy in the third case may have prevented the fulfillment of the patient’s best interest due to the lack of awareness among physicians regarding the objective of palliation. Palliative care is gradually spreading in Japan, and neonatal end-of-life healthcare guidelines were recently issued [18]. Yet, when deciding on a treatment strategy, participants indicated that since even one treatment can have multiple objectives to those involved, the degree of efficacy and side effects for each objective, as well as the possibility of inconsistencies among the objectives, should be carefully considered. As in the second case, the possibility that a treatment would be in the patient’s best interest regardless of its efficacy or side effects must also be carefully considered. This is particularly important when making decisions on whether to carry out treatments about which the opinions of those involved are divided, such as the use of anti-microbial agents and artificial hydration and nutrition, as well as resuscitation. In the first case, for example, although the treatment being carried out when the patient suffocated in the end was unknown, DNAR intentions should have been reconsidered when she initially healed.

#### Uncertainty of therapeutic efficacy

Given the uncertainties surrounding therapeutic efficacy, participants recognized through each NSD that it is difficult to judge the futility of treatments. There were discrepancies in opinions among those involved, such as patients and families who expect no limits to healing and palliation by treatment, healthcare providers who attempt aggressive treatment until chances of recovery are near zero, and others involved who promptly give up on healing and palliation. As in the first case, life-sustaining measures, such as the use of artificial respirators, are a controversial issue in Japan. For example, while such measures are avoided in some cases, discontinuation is not regulated by laws. This leads to hesitation and situations where beneficial treatments are not carried out, or conversely, futile life-prolonging treatments are continued [19].

#### Imminent death

Japanese guidelines on withholding or discontinuing life-sustaining treatments are premised on the patient being in the terminal stage and in a condition where there is very little chance of improvement or long-term life prolonging [7,20,21]. Answers derived in the first and third forums also considered imminent death to be one of the criteria for futile life-prolonging treatments. In the second forum, the answer was that regardless of how imminent death was and the degree of healing possible, the bottom line was whether the treatment went against the patient’s intentions and best interest. Since the patient desired treatment in the second case, we could not evaluate the situation of when a patient does not desire treatment. Thus, regardless of how imminent death is, this answer suggests the possibility that the futility of a treatment should also be considered based on whether a patient refuses treatment, the best interest of the patient, and whether the advanced wishes of a patient in an incurable condition, such as a vegetative state or severe dementia, were known.

### Process of judging whether a life-prolonging treatment is futile

#### Informed consent

Here, we consider the process involved in judging treatment objectives, and whether their effects are in the best interest of the patient. NSD revealed that the patient’s intentions should come first, and that the consensus of those involved and social acceptance are also required. Not desiring treatment (a criterion for futile treatments) refers to situations such as when the patient refuses treatment, when the patient loses the ability to make decisions as in the first case, the existence of some type of advance directive regarding treatment, when intentions are unclear, or, as with the infant in the third case, when the patient never had the ability to make decisions. According to participants, what was lacking, first and foremost, was the adequate provision of information and support for decision making to patients with the ability to make decisions, and to families of patients without this ability. They strongly criticized the paternalism of healthcare professionals, and indicated that the expansion of amenities for informed consent is a social challenge.

#### Refusal of treatment

Although we could not address the situation arising when a patient refuses treatment, discrepancies between the opinions of those involved can potentially occur. One such example is when a patient with the ability to make decisions refuses life-prolonging treatment, but healthcare professionals and the family consider the decrease in QOL from treatment to be within the permissible range. While those involved should probably try to convince the patient to undergo treatment and discuss the patient’s desires and concerns, respecting the patient’s decision, not forcing the patient to undergo treatment, and accepting the idea that the treatment is not in the patient’s best interest given his/her refusal comports with the answers derived from NSD. In such cases, while the aspect of “refusal” is stronger than “futility,” “futile” because of “refusal” might be accepted depending on the degree to which those involved and society respect the patient’s self-determination.

#### Representation based on advance directives

When a patient loses the ability to make decisions as in the first case, the family’s opinion is given weight. It is generally considered ethically appropriate for the patient’s family to make decisions as the patient’s representative if advance directives exist, or if not, based on presumed intent and the patient’s best interest [22]. While participants had ethical concerns with the eldest daughter’s assertion that “I know what my mother’s wishes are. I want her to live as long as possible, for my sake as well as her own.” in the first case, they did not go as far as requiring advance directives or presumed intent. Notwithstanding, participants agreed that the eldest daughter desired the treatment for healing and palliation, and concluded that the final judgment of whether the treatment is futile should require the eldest daughter’s consent. Indeed, the daughter’s request cannot be considered inappropriate, particularly when considering the uncertainties of treatment and its effects. Although there is no law regulating representation rights in the healthcare setting in Japan, the family conventionally serves as the representative, and Japanese guidelines also suggest obtaining consent from those involved [7,20,21]. In the United States, where the hierarchy of representative rights among those involved is determined by family consent laws, if a family member living away from home requests that physicians perform life-prolonging treatment, there are views that there is little choice but to carry out the treatment when opinions differ among children [23]. Accordingly, consent should have been obtained from the eldest daughter as the patient’s representative, and as indicated in the second case study, healthcare workers should accept the eldest daughter’s feelings and support her so that she could make a decision from the patient’s standpoint. This also suggests that there will be a need to further examine and understand the concepts of advance directives and representation in Japan.

#### Representation based on best interest

When a patient never had the ability to make decisions in the first place, as in the third case, participants agreed that judgments should be made based on the patient’s best interest [22]. In the third case, participants agreed that the physician should have explained the treatment strategy to the parents and took into consideration the parents’ intentions. Yet, since the patient’s interests were considered from the healthcare professional standpoint, participants argued that healthcare professionals should discuss strategy with parents in the event that they request treatments that contradict the patient’s best interest. In the third case, since the parents followed the healthcare professional’s treatment strategy, no situation in which family and healthcare professionals have differing opinions was examined. Indeed, although objectively judging the best interests of a patient who has no intentions may be possible, if parents are in a position to represent the infant’s interests, then the parents’ decision should be respected. While cases exist in Japan of treating children after terminating parental rights when parents refuse beneficial treatments for their children, treatments are ordinarily carried out when life-sustaining treatments are requested. The guidelines of the Japan Pediatric Society stipulate that although guardians or healthcare staff can propose the withholding or discontinuing of life-sustaining treatments, discussions should be held with both parties until they agree, and treatment should be continued until then [18]. Thus, even when a patient lacks the ability to make decisions to begin with, healthcare professionals should provide recommendations on treatment strategy, accept the family’s emotions, support and respect their representative capacity, and should not discontinue treatment if it goes against the family’s will.

#### Healthcare team consensus and social acceptance

There were differing views between physicians and nurses in every case. Participants also agreed that more extensive discussions on treatment strategy and consensus building were necessary, although this seemed impossible based on personnel and time constraints. When differing opinions exist within the healthcare team or between the healthcare team and patient/family, support should be sought externally. This relates to obtaining social acceptance, one of the criteria of futile life-prolonging treatments. Participants indicated that patient consulting services serve as such a medium, while government guidelines suggest making use of the hospital ethics committee. In the three cases discussed herein, an ethics committee was not consulted and this was not suggested by participants either. Moreover, excluding cases in which parental rights are terminated, pre-rulings by courts are scarce in Japan, and thus participants did not raise the possibility of using court systems. Future challenges in Japanese healthcare include team medicine, patient support, ethics committee consultation, and pre-rulings by courts, and thus the enhancement of medical resources is necessary. On the other hand, resource constraints cannot be ignored, and the futility of treatments have, in some instances, been argued in connection with medical cost cutting [9]. However, the criterion of social acceptance, as determined by NSD, must be realized when withholding or discontinuing treatments when it is the patient’s intention and in the patient’s best interest. This is not the case, however, when judging whether a treatment is futile in light of the patient’s intentions or best interest. As discussed in the second forum, in some instances, a beneficial treatment desired by the patient is not covered by insurance or beds may not be available in the sterile room, and thus treatment cannot be provided. This implies that medical resources are a social constraint imposed separately from judgments of treatment futility.

Finally, we collectively evaluate NSD discussions and answers derived therefrom as well as limitations of our study. Discussions on medical futility in the United States center on the fact that it is probable and influenced by value judgments and the various objectives of those involved. It is also something that physicians cannot unilaterally judge, but rather is determined through discussions and consensus among those involved. In this context, resource availability is considered a separate issue from futility [9]. Even participants who were unfamiliar with such arguments came to essentially the same conclusion through NSD. By expressing the objective of healthcare and the requirement of social acceptance, participants were also able to elucidate issues related to the awareness of those involved and the medical environment. Answers obtained from NSD are similar to the end-of-life guidelines in Japan in that they rely on achieving a consensus among those involved. However, rather than being limited to the terminal stage, the objective of treatment, its effects, and benefits should be more specifically discussed with the patient’s intentions as the foremost consideration. The family’s intentions come next, and the healthcare professionals should not be making the final decision.

As discussed above, however, the results of the NSD forums need further examination regarding several points: considering the futility of a treatment based on a patient’s (advanced) will in the context of an incurable condition regardless of how imminent death is; accepting futility due to a patient’s refusal; implementing advance directives and representation; handling a situation in which family and healthcare professionals have differing opinions on the treatment of a patient has no intentions; and adopting effective measures for obtaining social acceptance when discontinuing treatments according to a patient’s intention and best interest.

The NSD method used in this study was qualitative with a limited number of participants who were non-randomly selected. So they could not represent any groups in society. NSD discussions were also limited by the cases discussed, current situation, and facilitation. The participants, however, were serious about collaboratively deliberating on the issue. The results of their deliberation should therefore be respected in the same way as results in sorts of forums for participatory technology assessment with a limited number of non-randomly selected participants.

In order to reduce bias in results of each NSD forum, we also conducted three forums in different cities, compared and summarized their results, and examined them by reviewing relevant literature. Although we believe that our summary of the study results is appropriate, additional methods could have been planned to improve credibility, such as transcribing IC records of the forums into verbatim texts as well as sending an interim summary of each forum to its participants for authorization. We, in fact, sent a research report and a draft manuscript to participants who had shown willingness to receive the report on their consent forms at the end of the funding period (March 2012). None of the participants disagreed. Our study using the NSD method was conducive to generating appropriate answers to the question “What is life-prolonging treatment” and elucidating problems in practice.

## Conclusions

This small study contributed to elucidating the conditions that underlie futile life-prolonging treatments through NSD among participants of diverse backgrounds. Such treatments are those carried out for the sole purpose of prolonging life and are detrimental to the patient, and should be decided based foremost on the patient’s lack of desire for treatment, the consensus of those involved, and through social acceptance. Current issues that surround futile life-prolonging treatments in Japan were also elucidated through NSD, including an awareness of the objective of treatments and their effects, informed consent, advance directives, decision making by representatives, methods of social approval, and medical environment.

## Competing interests

The authors declare that they have no competing interests.

## Authors’ contributions

All authors participated in the design of the study. KA coordinated and facilitated the forums and drafted the manuscript. AA kept the record of the forums and helped to coordinate and facilitate them and draft the manuscript. SB coordinated a forum and helped to facilitate it. All authors read and approved the final manuscript.

## Pre-publication history

The pre-publication history for this paper can be accessed here:

http://www.biomedcentral.com/1472-6939/14/51/prepub

## References

[B1] Otani I ‘Good manner of dying’ as a normative concept: ‘Autocide’, ‘granny dumping’ and discussions on euthanasia/death with dignity in Japan Int J Jpn Sociol 2010 14 1 49 63 10.1111/j.1475-6781.2010.01136.x

[B2] Otake T A more dignified way to die: the hospice movement in Japan Jpn Times Online 2005 http://www.japantimes.co.jp/text/fl20051023x2.html

[B3] Shumatsuki Iryo no Arikata ni kansuru Kondankai [Commission on End-of-life Care] Shumatsuki Iryo no Arikata ni kansuru Kondankai Hokokusho [Report of the commission on end-of-life care] 2010 http://www.mhlw.go.jp/stf/shingi/2r9852000000yp23-att/2r9852000000yp3k.pdf

[B4] Nihon Songenshi Kyokai [Japan Society for Dying with Dignity Homepage] http://www.songenshi-kyokai.com/

[B5] Takano R Kokyuki hazushi ishi fukiso; “Satsujinzai seiritsu sezu” Toyama/Imizu [The doctor disconnecting respirators was not prosecuted; “It does not constitute homicide” in Imizu, Toyama] Asahi Shimbun December 22, 2009 39

[B6] Shumatsuki Iryo no Kettei Process no Arikata ni kansuru Kento-kai [Panel on the decision process for end-of-life care] Shumatsuki Iryo no Kettei Process ni kansuru Guideline [Guidelines regarding the decision process for end-of-life care] 2007 http://www.mhlw.go.jp/shingi/2007/05/dl/s0521-11a.pdf

[B7] Shumatsuki Iryo no Kettei Process no Arikata ni kansuru Kento-kai [Panel on the decision process for end-of-life care] Shumatsuki Iryo no Kettei Process ni kansuru Guideline Kaisetsuhen [A commentary on the guidelines regarding the decision process for end-of-life care] 2007 http://www.mhlw.go.jp/shingi/2007/05/dl/s0521-11b.pdf

[B8] Enmei chushi, team de handan; Shumatsuki teigi wa sakiokuri; Kuni ga hatsu shishin Enmei chushi, team de handan; Shumatsuki teigi wa sakiokuri; Kuni ga hatsu shishin [Team decides to stop life-prolongation; The first national guidelines postpone definition of end-of-life] Asahi Shimbun April 10, 2007 1

[B9] Helft PR Siegler M Lantos J The rise and fall of the futility movement N Engl J Med 2000 14 4 293 296 10.1056/NEJM200007273430411 10911014

[B10] Kadooka Y Asai A Aizawa K Bito S Japanese healthcare workers’ attitudes towards administering futile treatments: a preliminary interview-based study Eubios J Asian Int Bioethics 2011 14 4 131 136

[B11] Philosophisch-Politische Akademie [The Philosophical-Political Academy] http://www.philosophisch-politische-akademie.de/

[B12] Society for the Furtherance of the Critical Philosophyhttp://www.sfcp.org.uk/

[B13] Griessler E Littig B Husing B Zimmer R Santos D Munoz E Ponce G Gronke H Dordoni P Final Report Increasing Public Involvement in Debates on Ethical Questions of Xenotransplantation 2004: European Commission DG XII http://www.ihs.ac.at/departments/soc/xeno-pta/final.pdf

[B14] Horie T Kokyoteki taiwa no hoho: Zaitaku ni okeru iryo koi o tema ni shita taiwa compotents no kokoromi [A method of public dialogues: a trial of a dialogue complex on the theme of home medical treatment] Rinsho Communication no Model Kaihatsu to Jissen [Model Development and Practice for Clinical Communication], Research Report 2002–2003 Head Investigator: Washida K 2004: Science and Technology Policy Proposal, MEXT Fund for Coordinating Science and Technology Development 34 46

[B15] Griessler E Pichelstorfer A Horie T Gronke H Littig B Szyma AD in corporation with Neosokratische Dialoge zu ethischen Fragen genetischer Beratung in Oesterreich, Deutschland und Japan [Neo-Socratic Dialogues concerning ethical questions of genetic counseling in Austria, Germany and Japan] 2009 http://www.ihs.ac.at/steps/gendialog/english/NSD_Endbericht09.pdf

[B16] Aizawa K Asai A Kobayashi Y Hoshiko K Bito S A Neo-Socratic Dialogue for developing a mutual understanding of rights and responsibilities in the healthcare system Contemp Appl Philos 2010 14 10001 10016 http://openjournals.kulib.kyoto-u.ac.jp/ojs/index.php/cap/article/viewFile/63/17

[B17] Aizawa K Asai A Kobayashi Y Hoshiko K Bito S Neo-Socratic Dialogue on fairness in the healthcare system Eubios J Asian Int Bioethicsin press

[B18] Nihon Shonika Gakkai Rinri Iinkai Shoni Shumatsuki Iryo Guideline Working Group [The Japan Pediatric Society Ethics Committee’s Working Group on Pediatric End-of-life Guidelines] Jutoku na shikkan o motsu kodomo no iryo o meguru hanashiai no guideline (2012 nen 4 gatsu 20 ka rinri iinkai shoninban) [Guidelines for discussions on healthcare for children with severe diseases (A version approved by the Ethics Committee on April 20, 2012)] J Japan Pediatr Soc 2012 14 10 1 16

[B19] Aita K Enmei Iryo to Rinsho Genba: jinko kokyuki to iro no iryo rinrigaku Life-sustaining treatment and clinicians in Japan: medical ethics of mechanical ventilation and PEG tube feeding 2011 Tokyo: University of Tokyo Press 142 144 211–213

[B20] Nihon Ishikai [Japan Medical Association] Shumatsuki iryo no guideline 2009 [Guidelines on end-of-life care 2009] Grand Des 2009 14 67 70 http://dl.med.or.jp/dl-med/nichikara/gd2009.pdf

[B21] Zennihon Byoin Kyokai Shumatsuki Iryo ni kansuru Guideline Sakutei Iinkai [All Japan Hospital Association’s Planning Panel for Guidelines on End-of-life Care] Shumatsuki iryo ni kansuru guideline [Guidelines on end-of-life care] 2009 http://www.ajha.or.jp/topics/info/pdf/2009/090618.pdf

[B22] Lo B Resolving Ethical Dilemmas: A guide for clinicians 2009 4 Philadelphia, PA: Wolters Kluwer Health/Lippincott Williams & Wilkins 88 106

[B23] Molloy DW Clarnette RM Braun EA Eisemann MR Sneiderman B Decision making in the incompetent elderly: “The daughter from California syndrome J Am Geriatr Soc 1991 14 4 396 399 2010590 10.1111/j.1532-5415.1991.tb02907.x

